# Mitochondrial pathways of copper neurotoxicity: focus on mitochondrial dynamics and mitophagy

**DOI:** 10.3389/fnmol.2024.1504802

**Published:** 2024-12-05

**Authors:** Michael Aschner, Anatoly V. Skalny, Rongzhu Lu, Airton C. Martins, Yousef Tizabi, Sergey V. Nekhoroshev, Abel Santamaria, Anton I. Sinitskiy, Alexey A. Tinkov

**Affiliations:** ^1^Department of Molecular Pharmacology, Albert Einstein College of Medicine, Bronx, NY, United States; ^2^Institute of Bioelementology, Orenburg State University, Orenburg, Russia; ^3^Center of Bioelementology and Human Ecology, IM Sechenov First Moscow State Medical University (Sechenov University), Moscow, Russia; ^4^Department of Medical Elementology, Peoples’ Friendship University of Russia (RUDN University), Moscow, Russia; ^5^Department of Preventive Medicine and Public Health Laboratory Science, School of Medicine, Jiangsu University, Zhenjiang, China; ^6^Department of Pharmacology, Howard University College of Medicine, Washington, DC, United States; ^7^Problem Research Laboratory, Khanty-Mansiysk State Medical Academy, Khanty-Mansiysk, Russia; ^8^Facultad de Ciencias, Universidad Nacional Autónoma de México, Mexico City, Mexico; ^9^Laboratorio de Nanotecnología y Nanomedicina, Departamento de Atención a la Salud, Universidad Autónoma Metropolitana-Xochimilco, Mexico City, Mexico; ^10^Department of Biochemistry, South Ural State Medical University, Chelyabinsk, Russia; ^11^Laboratory of Ecobiomonitoring and Quality Control and Department of Physical Education, Yaroslavl State University, Yaroslavl, Russia

**Keywords:** copper, mitophagy, mitochondrial fusion, fission, cuproptosis

## Abstract

Copper (Cu) is essential for brain development and function, yet its overload induces neuronal damage and contributes to neurodegeneration and other neurological disorders. Multiple studies demonstrated that Cu neurotoxicity is associated with mitochondrial dysfunction, routinely assessed by reduction of mitochondrial membrane potential. Nonetheless, the role of alterations of mitochondrial dynamics in brain mitochondrial dysfunction induced by Cu exposure is still debatable. Therefore, the objective of the present narrative review was to discuss the role of mitochondrial dysfunction in Cu-induced neurotoxicity with special emphasis on its influence on brain mitochondrial fusion and fission, as well as mitochondrial clearance by mitophagy. Existing data demonstrate that, in addition to mitochondrial electron transport chain inhibition, membrane damage, and mitochondrial reactive oxygen species (ROS) overproduction, Cu overexposure inhibits mitochondrial fusion by down-regulation of Opa1, Mfn1, and Mfn2 expression, while promoting mitochondrial fission through up-regulation of Drp1. It has been also demonstrated that Cu exposure induces PINK1/Parkin-dependent mitophagy in brain cells, that is considered a compensatory response to Cu-induced mitochondrial dysfunction. However, long-term high-dose Cu exposure impairs mitophagy, resulting in accumulation of dysfunctional mitochondria. Cu-induced inhibition of mitochondrial biogenesis due to down-regulation of PGC-1α further aggravates mitochondrial dysfunction in brain. Studies from non-brain cells corroborate these findings, also offering additional evidence that dysregulation of mitochondrial dynamics and mitophagy may be involved in Cu-induced damage in brain. Finally, Cu exposure induces cuproptosis in brain cells due mitochondrial proteotoxic stress, that may also contribute to neuronal damage and pathogenesis of certain brain diseases. Based on these findings, it is assumed that development of mitoprotective agents, specifically targeting mechanisms of mitochondrial quality control, would be useful for prevention of neurotoxic effects of Cu overload.

## 1 Introduction

Copper (Cu) is an essential element involved in a plethora of metabolic processes through its structural, catalytic (Tsang et al., 2021) and signaling functions (Ackerman and Chang, 2018). Adequate Cu concentration is required for brain development and functioning due to its involvement in energy and redox metabolism, regulation of iron balance and neurotransmission (Scheiber et al., 2014), as well as neuroproteostasis (Opazo et al., 2014). Due to multiple functions of Cu in brain, its deficiency may induce neuronal damage (Zatta and Frank, 2007), contributing to certain neurological disorders including myeloneuropathy (Altarelli et al., 2019).

In contrast, Cu overload may also induce adverse effects in brain due to its neurotoxic injury. The existing data demonstrate that Cu overload is involved in neurodegeneration (Anandhan et al., 2015; Giampietro et al., 2018; Chen et al., 2023). Specifically, meta-analysis of epidemiological data demonstrated that high circulating Cu level is associated with Alzheimer’s disease (AD) (Squitti et al., 2021). Epidemiological observations also link excessive Cu levels with ischemic stroke (Zhang et al., 2021), depression (Ni et al., 2018), and epilepsy (Chen et al., 2024). Cu overload may also contribute to neurodevelopmental disorders including autism spectrum disorder (ASD) (Santos et al., 2019), similar to what is reported for lead (Pb) (Tizabi et al., 2023), as well as in Huntington disease (HD) (Tizabi et al., 2024). In addition to Cu overexposure, altered Cu transportation and storage may induce cellular Cu overload leading to adverse neurological effect, like that observed in Wilson’s disease (WD) due to mutation in ATP7B gene (Członkowska et al., 2018). Therefore, tight regulation of Cu trafficking in brain is required for prevention of its toxicity (Lutsenko et al., 2010).

Epidemiological data corroborate experimental evidence demonstrating the role of Cu exposure in behavioral disorders. Specifically, Cu exposure in laboratory rodents induced anxiety (Abbaoui et al., 2017), depression (Lamtai et al., 2022), impaired locomotor performance (Abbaoui and Gamrani, 2018; Kalita et al., 2020). Furthermore, learning and memory deficits are also observed in laboratory animals upon Cu exposure (Pal et al., 2013; Lamtai et al., 2020).

The role of Cu overexposure in doses exceeding the physiological demands in neuropathology is also supported by the observed neuroprotective effects Cu-targeting therapies, including metal chelation and addressing molecular mechanisms of Cu neurotoxicity. Specifically, it has been demonstrated that Cu chelators bathocuproine disulphonate significantly reduced neuronal damage in hippocampus of rats following intra-hippocampal injections of Cu (Armstrong et al., 2001). It has been also demonstrated that native metallothionein is capable of preventing Cu-mediated amyloid *β* aggregation and subsequent toxicity in cultured cortical neurons (Chung et al., 2010). Cu chelation also ameliorates Cu toxicity in brain astrocytes (Bulcke et al., 2015). Correspondingly, Cu chelation possessed neuroprotective effects in Cu-induced Alzheimer’s disease model (Zhao et al., 2021), dementia (Tan et al., 2019), or autoimmune encephalomyelitis (Offen et al., 2004).

Adverse effects of Cu on brain function are mediated by a variety of toxicological mechanisms. Being a redox-active metal, Cu overload results in excessive production of reactive oxygen species (ROS) through Fenton reaction and oxidative stress in neurons and astrocytes (Witt et al., 2021; Lu et al., 2022). Through induction of oxidative stress and modulation of redox-sensitive transcription factors including nuclear factor kappa B (NF-κB), Cu promotes neuroinflammation (Hu et al., 2014). Cu overload induces neuronal death through multiple pathways including not only apoptosis (Lu et al., 2022), but also cuproptosis (Zhang K. et al., 2023; Zhang Y. et al., 2023) and ferroptosis (Wang et al., 2024; Wang et al., 2024a,b). Furthermore, Cu ions promote oligomerization and aggregation of both amyloid beta (Aβ) (Karr and Szalai, 2008; Jin et al., 2011) and alpha-synuclein (*α*-Syn) (Wright et al., 2009), thus contributing to neurodegeneration. In addition to Ab oligomerization, Cu exposure may aggravate Aβ toxicity through modulation of its production and clearance mechanisms (Singh et al., 2013). Studies have highlighted the role of epigenetic mechanisms including modulation of microRNA (miR) expression (Hsu et al., 2019), DNA methylation (Sarode et al., 2021), and histone modifications (Alsadany et al., 2013) in Cu neurotoxicity. Further understanding of the molecular mechanisms underlying Cu neurotoxicity is essential for estimation of therapeutic targets for Cu-associated neurological disorders (Feng et al., 2023).

Earlier studies suggested the potential role of mitochondria as a target for Cu neurotoxicity (Rossi et al., 2004). Specifically, brain mitochondria are characterized by high susceptibility to Cu toxicity (Borchard et al., 2018), and this interplay may contribute to Cu-associated brain disorders (Arciello et al., 2005). The majority of studies have used mitochondrial transmembrane potential as a routine marker for assessment of mitochondrial dysfunction in Cu-induced neurotoxicity (Shi et al., 2008; Goto et al., 2020; Witt et al., 2021), as well as the impact of Cu overload on brain mitochondrial electron transport chain (ETC) (Belyaeva et al., 2012) and ROS production. However, the interference of Cu with mitochondrial dynamics and mitophagy that are involved in mitochondrial quality control (Ashrafi and Schwarz, 2013) and play a significant role in brain physiology (Khacho and Slack, 2018) and pathology (Burté et al., 2015) has been insufficiently addressed.

It has been demonstrated that mitochondrial protection plays a significant role in neuroprotection against adverse effects of Cu in brain (Borchard et al., 2021; Goma et al., 2021). Furthermore, improvement of mitochondrial functioning was shown to be an effective approach against Aβ toxicity in a non-transgenic (Lahmy et al., 2015) and transgenic Alzheimer’s disease models (An et al., 2019). Correspondingly, restoration of mitochondrial functioning and biogenesis is considered a potential approach to Parkinson’s disease treatment (Xi et al., 2018; Zheng et al., 2023). Targeting mitochondrial dysfunction significantly contributed to reduction of apoptosis in hippocampus, thus preventing depression development (Chen et al., 2017). Together with the data on the role of Cu exposure in mitochondrial dysfunction, these findings indicate that further understanding of the interference between Cu neurotoxicity and mitochondrial functioning may reveal additional therapeutic targets for treatment of Cu-induced neuropathology including neurodegeneration including Alzheimer’s and Parkinson’s diseases.

Therefore, the objective of the present narrative review was to discuss the role of mitochondrial dysfunction in Cu-induced neurotoxicity with a special focus on the influence of Cu on brain mitochondrial fusion and fission, as well as mitochondrial clearance by mitophagy.

## 2 A brief introduction into cu trafficking

Due to potential toxicity of Cu in its free form, transport of Cu in the cells including neurons is strictly regulated. The molecular mechanisms of intracellular Cu trafficking (Lutsenko, 2016; Lutsenko et al., 2024) including that in brain (Lutsenko et al., 2010; Wen et al., 2021) are highlighted in a number of excellent reviews, and thus a brief overview of Cu handling mechanisms with a focus on its mitochondria will be provided. Cu is transported into the cell through high-affinity transporter, copper transporter 1 (Ctr1), that is located on the plasma membrane and transports Cu(I) (Walke et al., 2022). It is also suggested that Cu(II) may be transported into the cell through low-affinity transporters like divalent metal transporter 1 (DMT1) (Lutsenko, 2016).

Once entering the cytoplasm Cu is bound to low-molecular weight ligands including GSH or to cytosolic chaperones that promote its transport to Cu-binding proteins (Cobine et al., 2021). Specifically, Cu chaperone CCS performs Cu delivery to SOD1 for its maturation (Casareno et al., 1998). ATOX1 transports Cu to copper-transporting P-type ATPases, namely ATP7A in the brain, transporting Cu into trans-Golgi network for its incorporation into cuproproteins including multi-Cu oxidases (Gromadzka et al., 2020). It has been also proposed that ATOX1 binds TRAF4 in a Cu-dependent manner for subsequent nuclear translocation and regulation of downstream gene expression (Das et al., 2019).

Mitochondria require substantial amounts of Cu due to the role of the latter in cytochrome c oxidase (CCO), being a final electron acceptor in the mitochondrial electron transport chain. Cu enters mitochondria as an anionic non-protein copper ligand (CuL) or bound to GSH (Zischka and Einer, 2018). It has been demonstrated that SLC25A3 located on the mitochondrial inner membrane may also mediate Cu entrance into the mitochondrial matrix (Boulet et al., 2018), where it is stored as CuL (Zischka and Einer, 2018). In the intermembrane space, Cu chaperone COX17 delivers Cu to chaperone for synthesis of cytochrome C oxidase 1 (SCO1) and COX11. The latter two chaperones provide Cu for metalation of CuA and CuB sites of CCO, respectively, thus contributing to CCO complex formation (Cobine et al., 2006).

In case of cellular Cu overload, ATP7A is relocalized from trans-Golgi network to plasma membrane to facilitate Cu export from the cell, and excessive Cu is bound to metallothioneins (Cobine et al., 2021). Cu sensing in the cell and subsequent modulation of Cu handling proteins expression is mediated by transcription factors. Specifically, binding Cu to MTF1 and SP1, the key Cu sensors in the cell, modulates expression of Ctr1, ATP7A/B, and SLC25A3 (Fitisemanu and Padilla-Benavides, 2024). In addition, Nrf1 and Nrf2 are also involved in modulation of Cu transport through up-regulation of MT expression in response to Cu-induced oxidative stress (Song et al., 2014). Taken together, Cu transport in the cell, including mitochondria, is tightly regulated through a network of transport proteins and chaperones, while dysregulation of these mechanisms, especially along with Cu exposure, results in Cu toxicity.

## 3 Cu-induced mitochondrial dysfunction

### 3.1 The role of mitochondrial ROS overproduction and mitochondrial damage in cu exposure-related neurotoxicity

Existing data demonstrate high susceptibility of neuronal mitochondria to Cu neurotoxicity, as mitochondrial membrane damage and subsequent dysfunction in neuroblastoma SHSY5Y cells has been documented even at low doses of Cu. Cu-induced mitochondrial damage characterized by cristae thinning was shown to be mediated by targeting free mitochondrial protein thiols, whereas ROS overproduction occurred at later stages only in damaged mitochondria (Borchard et al., 2018). Reduction of mitochondrial transmembrane potential and mitochondrial swelling in Cu^2+^-exposed human neuroblastoma SH-SY5Y cells, commonly used as an *in vitro* model of neuronal functioning, were associated with apoptosis and endoplasmic reticulum stress, altogether resulting in a significant reduction of cell viability (< 50%) after 24 h of exposure to at least 200 μM Cu (Chakraborty et al., 2022). It has been also noted that neuroprogenitor cells from patients with PARK2 mutations are characterized by higher susceptibility to mitochondrial fragmentation and subsequent mitochondrial dysfunction due to ROS overproduction following 48 h of Cu exposure at 100 μM (Aboud et al., 2015), indicative of the potential role of Cu-induced mitochondrial damage in PD pathogenesis.

Furthermore, reduction of MES23.5 dopaminergic cell viability induced by 24-h of exposure to at least 400 μM Cu was also associated with reduction of mitochondrial membrane potential (Shi et al., 2008). Noteworthy, mitochondrial dysfunction evidenced by a decrease in mitochondrial membrane potential is observed upon exposure to non-cytotoxic Cu doses in MES23.5 dopaminergic cells (Shi et al., 2008). Paris et al. (2009) demonstrated that Cu-dopamine complex formed in dopaminergic neurons induced mitochondrial dysfunction and mitophagy, leading to caspase-independent cell death (Paris et al., 2009). In addition, Cu promoted 6-hydroxydopamine autooxidation, resulting in oxidative stress in rat brain mitochondria (Cruces-Sande et al., 2017). However, some studies demonstrated that Cu-induced ROS production and oxidative stress in dopaminergic neurons is generally localized in the cytoplasm rather than in mitochondria (Anandhan et al., 2015).

Cu-induced alterations in mitochondrial redox homeostasis also included a variety of mechanisms. Specifically, administration of Cu and cholesterol-rich diet significantly affected mitochondrial membrane lipid composition, resulting in higher cholesterol-to-phospholipid ratio with subsequent increase in membrane rigidity and permeability, also accompanied by depletion of mitochondrial reduced glutathione (GSH) pool (Arnal et al., 2013). In addition, Cu exposure significantly reduced glutaredoxin (GRX1) activity in neuroblastoma cells due to Cu-binding activity of the latter, whereas Grx1 overexpression significantly reduces mitochondrial Cu accumulation despite the overall higher cellular Cu content and copper transport protein 1 (Ctr1) up-regulation (De Benedetto et al., 2014). It has also been hypothesized that Cu exposure during glucose deprivation induces release of Ca^2+^ and Zn^2+^ from intracellular stores, their transport to mitochondria, and subsequent mitochondrial toxicity (Isaev et al., 2016).

Cu-induced mitochondrial damage is also aggravated by other neurotoxic agents. Specifically, H_2_S also promoted Cu neurotoxicity in SH-SY5Y cells through aggravation of mitochondrial dysfunction as evidenced by a decline in mitochondrial membrane potential and reduction of ATP production due to increased mitochondrial ROS production (Goto et al., 2020). Neurotoxic effects of Cu were heightened by co-exposure with homocysteine, that up-regulated ROS generation and mitochondrial dysfunction, leading to cytochrome c leakage and nuclear translocation of apoptosis-inducing factor (AIF), ultimately resulting in SH-SY5Y cell apoptosis (Hirashima et al., 2010). It is also notable that Pb can activate microglia in APP/PS1 mice through increasing translocation of mitochondrial copper transporter cytochrome c oxidase copper chaperone (COX17) with subsequent mitochondrial Cu^2+^ accumulation and induction of mitochondrial dysfunction (Huang D. et al., 2024; Huang Q. et al., 2024).

Adverse effects of Cu exposure may also target astrocyte mitochondria. Specifically, it has been proposed that reduction of mitochondrial membrane potential was essential for Cu-induced astrocyte death, whereas neuronal damage was more related to oxidative and nitrosative stress (Reddy et al., 2008). At the same time, it has been demonstrated that neurons were more sensitive to Cu toxicity than astrocytes, as exposure to 20 μM CuSO_4_ significantly increased LDH release by 40% from cultured astrocytes only at 24 h, while similar effect in a neuronal culture was observed already after 14 h of exposure to a twofold lower dose (10 μM CuSO_4_) (Reddy et al., 2008). A more recent study also demonstrated that Cu exposure induced a dose-dependent decrease in mitochondrial membrane potential along with mitochondrial ROS overproduction in astrocytes characterized by a 30% reduction in cell viability upon exposure to 250 μM CuSO4 for 48 h (Witt et al., 2021). It is assumed that these effects may result in mitochondrial dysfunction with a subsequent cytochrome c release, caspase 3 activation, and apoptosis (Chen et al., 2008). In addition, Cu significantly increased sensitivity of astrocytes to agents inducing mitochondrial membrane potential reduction and mitochondrial ROS generation (Gyulkhandanyan et al., 2003).

Taken together, these findings demonstrate high susceptibility of brain mitochondria to Cu toxicity, and that Cu-induced mitochondrial dysfunction may even precede Cu-induced ROS overproduction and oxidative stress. In addition to neurons, Cu exposure may also induce mitochondrial dysfunction in glial cells, thus disturbing its functions.

### 3.2 Cu-induced alteration of mitochondrial enzymes

Given the role of electron transport chain in the formation of mitochondrial membrane potential (Zorova et al., 2018), a number of studies revealed adverse effects of Cu on respiratory complexes and its role in Cu cytotoxicity. Exposure of SH-SY5Y cells to Cu significantly reduced cell viability through mitochondrial Cu accumulation with subsequent ROS overproduction and inhibition of mitochondrial electron transport chain complexes with Complex I being the most sensitive (Arciello et al., 2005). In an earlier study, Heron et al. (2001) demonstrated that Cu exposure reduced mitochondrial electron transport in rat striatal mitochondria with inhibition of NADH-dependent lactate dehydrogenase (LDH) activity and FAD-dependent monoamine oxidase A (MAO-A) activity in a dose-dependent manner (Heron et al., 2001). A significant inhibitory effect of Cu^2+^ on pyruvate dehydrogenase and *α*-ketoglutarate dehydrogenase activity in cortical neuronal cells (Sheline and Choi, 2004) due to ROS overproduction was demonstrated (Sheline and Wei, 2006).

In addition to ionic Cu^2+^, copper oxide nanoparticles (CuONPs) also induced mitochondrial dysfunction with inhibition of mitochondrial dehydrogenases aldehyde dehydrogenase (ALDH2) and glutamate dehydrogenase (GDH), both of which contribute to CuONPs-induced neuronal damage and neurobehavioral deficits along with neuronal oxidative stress (Goma et al., 2021).

Long-Evans Cinnamon rats, used as a model of WD with Cu overload, are characterized by down-regulation of genes involved in mitochondrial electron transport chain functioning, including succinate dehydrogenase complex assembly factor (Sdhaf2) and NADH dehydrogenase [ubiquinone] 1 beta subcomplex subunit 7 (Ndufb7) (Lee et al., 2013). Proteomic analysis of mouse cortex demonstrated that long-term low-dose Cu exposure results in significant alteration of mitochondrial proteome with down-regulation of proteins involved in mitochondrial functioning, including isocitrate dehydrogenase [NAD(+)] 3 catalytic subunit alpha (IDH3A), ATP synthase subunit delta (ATPD), N-ethylmaleimide sensitive factor, vesicle fusing ATPase (NSF), as well as 75 kDa (GRP75) and 78 kDa glucose-regulated proteins (GRP78). The authors propose that, in addition to mitochondrial dysfunction, down-regulation of mitochondrial GRP75 and GRP78 may contribute to apoptosis and endoplasmic reticulum stress, both contributing to Cu neurotoxicity (Lin et al., 2016).

Therefore, inhibition of brain mitochondrial electron transport chain may mediate a decline in mitochondrial membrane potential and contribute to Cu neurotoxicity. Inhibitory effects of Cu on electron transport chain enzymes may result from Cu-induced ROS overproduction as well as modulation of transcription factors activity like NRF2 (Fitisemanu and Padilla-Benavides, 2024). As a number of electron transport chain enzymes are encoded by mitochondrial genome, that contains 37 genes (Chinnery and Hudson, 2013), it is also questionable whether Cu may modulate expression of mitochondrial genes.

### 3.3 Interactive effects of cu and Aβ in mitochondrial dysfunction

Although Cu is considered a factor promoting Aβ aggregation, thus contributing to the development of AD (Hung et al., 2010), several studies also demonstrated that Cu significantly increases toxic effects of Aβ and α-syn in mitochondria.

Specifically, it has been demonstrated that Aβ_1-42_-induced inhibition of mitochondrial cytochrome c oxidase is dependent on availability of Cu^2+^ (Crouch et al., 2005) and this process is independent of H_2_O_2_ generation, hypothetically involving formation of amyloid-beta-methionine radical (Crouch et al., 2006). Furthermore, it is assumed that Aβ-Cu(II) may take part in redox reaction with pyruvate, as well as promote H_2_O_2_-mediated oxidation of pyruvate, thus impairing electron transport chain of mitochondria and reducing ATP generation (Jiang et al., 2007). These findings agree with results of another study demonstrating neuroprotective effect of pyruvic acid against Cu^2+^/Zn^2+^-induced neurotoxicity in GT1-7 cells through reduction of mitochondrial damage and cytochrome c release (Tanaka et al., 2018). Concomitantly, treatment with ruthenium(II) polypyridyl complexes significantly ameliorated mitochondrial dysfunction induced by Aβ-Cu^2+^ in SH-SY5Y cells through Cu^2+^ chelation and *π*-π stacking interaction with Aβ protein, thus limiting Aβ aggregation (Peng et al., 2021).

Significant trilateral interplay between Cu exposure, Aβ, and mitochondrial dysfunction was demonstrated in AD models. Specifically, exposure to Cu with drinking water (0.13 ppm CuCl2) for 12 months in triple-transgenic 3xTg-AD mice characterized by three Alzheimer’s disease-specific mutations (APP Swedish, MAPT P301L, PSEN1 M146V), resulted in brain oxidative stress, synaptic dysfunction, Aβ accumulation, spatial memory impairment, as well as a significant disruption of both mitochondrial and nuclear proteome. Cu exposure down-regulated 10 and up-regulated 14 mitochondrial proteins involved in energy metabolism, cytoskeleton formation, DNA damage and apoptosis, and tricarboxylic acid cycle (TCA) cycle, together with reduced ATP content being indicative of mitochondrial dysfunction (Yu et al., 2018). Cu^2+^ exposure also increased Aβ-induced mitochondrial membrane damage, mitochondrial swelling, reduction of mitochondrial transmembrane potential, depletion of mitochondrial GSH content with ROS overproduction, along with reduction in ATP production, altogether contributing to aggravated spatial learning and memory deficits (Behzadfar et al., 2017). Cu exposure significantly reduced mitochondrial membrane potential and increased cytochrome c release from mitochondria, subsequently leading to apoptosis in APPsw cells, a cellular model of AD (Zhao et al., 2013).

Potentiation of Aβ-induced microglial cell BV-2 activation by Cu^2+^ is mediated by activation of NF-κB signaling and mitochondrial ROS production, ultimately increasing cell death in a culture of primary hippocampal neurons exposed to microglia-conditioned medium (Yu et al., 2015). Hu et al. (2014) demonstrated that up-regulation of NF-κB signaling leading to subneurotoxic Cu-induced microglial activation was dependent on mitochondrial superoxide generation rather than increased NADPH-oxidase activity (Hu et al., 2014).

These findings demonstrate that Cu exposure potentiates A*β*-induced mitochondrial dysfunction both *in vitro* and *in vivo*, thus increasing its toxicity and promoting AD development.

## 4 Cu-induced alterations in mitochondrial dynamics and mitophagy

### 4.1 A brief overview of mitochondrial dynamics and mitophagy

#### 4.1.1 Mitochondrial fusion and fission

Mitochondria are highly dynamic organelles that undergo fission and fusion processes, and the balance between fission and fusion cycles is defined as mitochondrial dynamics (Yang D. et al., 2021; Yang F. et al., 2021). Mitochondrial dynamics control the shape, size and mitochondrial functioning to meet metabolic demands of the cells (Fenton et al., 2021). The balance between mitochondrial fission and fusion has a significant impact on cellular bioenergetics. Specifically, fused mitochondria are characterized by high respiratory activity corresponding to increased energy demands, whereas fragmented mitochondria generated by fission have low respiratory activity that may be observed in resting cells (Westermann, 2012).

Mitochondrial fission is a process of division of a single organelle into two daughter mitochondria (Tilokani et al., 2018). It is essential for cellular division by providing growing cells with adequate number of mitochondria, as well as for redistribution of mitochondria within the cell (Al Ojaimi et al., 2022). Mitochondrial fission is also involved in mitochondrial quality control by producing dysfunctional daughter mitochondrial unit that is subsequently removed by autophagy (Twig and Shirihai, 2011). Briefly, mitochondrial fission involves participation of endoplasmic reticulum that drives pre-constriction of mitochondria, and dynamin-related GTPase dynamin-related protein 1 (Drp1) that promotes further conformational changes leading to fission (Tilokani et al., 2018). This process is dependent on recruitment of Drp1 to the outer mitochondrial membrane, that is mediated by membrane proteins like mitochondrial fission protein 1 (Fis1) and mitochondrial fission factor (Mff) (Suárez-Rivero et al., 2016).

In contrast to mitochondrial fission, fusion results in the formation of a single elongated mitochondrion from two distinct mitochondria. It is essential for the exchange of genetic material between mitochondria and maintenance of mitochondrial function (Chen and Chan, 2010). In addition, mitochondrial fusion may prevent elimination of damaged mitochondria by mitophagy due to dilution of impaired respiratory components (Twig and Shirihai, 2011). Mitochondrial fusion involves fusion of the outer and inner mitochondrial membranes that is mediated by mitofusins 1 and 2 (MFN1 and MFN2), and optic atrophy 1 protein (OPA1), respectively (Sabouny and Shutt, 2020).

In addition to mitochondrial fusion that contributes to improved mitochondrial activity, cells may also increase mitochondrial mass through biogenesis that is driven by peroxisome proliferator-activated receptor *γ*-coactivator-1α and β (PGC-1α and PGC-1β) (Ding et al., 2021). In addition, PGC-1α is involved in regulation of mitochondrial fusion and fission through modulation of *Mfn1*, *Mfn2*, and *Drp1* gene expression (Chen et al., 2022). Furthermore, mitochondrial turnover is also tightly associated with mitochondrial motility and its movement through the cytoskeleton by kinesin and dynein (Ni et al., 2015).

Adequate mitochondrial dynamics is essential for brain development and functioning (Khacho and Slack, 2018) through its impact on neurogenesis and neuronal plasticity (Bertholet et al., 2016). Correspondingly, alterations in mitochondrial dynamics are involved in pathogenesis of various brain diseases (Shen et al., 2023). Specifically, it has been demonstrated that impairments in mitochondrial fission and fusion are involved in neurodegeneration in AD, HD, and Parkinson’s disease (PD) (Yang D. et al., 2021; Yang F. et al., 2021). In addition, alterations in mitochondrial dynamics are associated with diabetic brain damage (Fernandes et al., 2020), cerebral ischemia (Kumar et al., 2016), neonatal hypoxic brain injury (Jones and Thornton, 2022).

Given the role of mitochondrial dynamics in maintenance of adequate mitochondrial function, it is not surprising that it can be affected by various stressors (Fu et al., 2019). Thus, in addition to energy stress, exposure to environmental toxicants like Cu may impair mitochondrial dynamics, hence contributing to cellular toxicity (Meyer et al., 2017).

#### 4.1.2 Mitochondrial autophagy (mitophagy)

Mitophagy is one of the forms of autophagy that selectively targets damaged mitochondria through formation of autophagosomes and its subsequent fusion with lysosomes for degradation and removal (Iorio et al., 2021). Mitophagy is a key component of mitochondrial quality control that removes damaged mitochondria, thus preventing its accumulation (Pickles et al., 2018). Given the role of mitophagy in mitochondrial clearance, mitochondrial membrane depolarization is considered the key trigger for mitophagy. Other factors inducing mitophagy include hypoxia, iron chelation, high glucose levels, and increased NAD^+^ levels (Killackey et al., 2020). Mitophagy is activated through distinct pathways, including non-receptor mediated mitophagy and receptor-mediated autophagy (Swerdlow and Wilkins, 2020).

Non-receptor-mediated mitophagy is a pathway of classical mitophagy that involves phosphatase and tensin homolog (PTEN)-induced kinase 1 (PINK1) and Parkin. Briefly, PINK1, containing a mitochondrial targeting sequence, is considered a mitochondrial damage sensor. In healthy mitochondria, PINK1 is transported to the inner mitochondrial membrane and subsequently degraded by presenilin-associated rhomboid-like protease (PARL) (Swerdlow and Wilkins, 2020). Mitochondrial damage associated with membrane depolarization results in inhibition of PINK1 translocation and its accumulation on the outer mitochondrial membrane, where it recruits Parkin (Lu et al., 2023). Activation of Parkin, a E3 ubiquitin ligase, promotes ubiquitination of outer mitochondrial membrane proteins including MFN1/2 and voltage-dependent anion channel 1 (VDAC1) (Wang et al., 2023). Mitochondrial protein phosphorylation and ubiquitination initiates recruitment of autophagy adaptors, including optineurin (OPTN) and nuclear dot protein 52 kDa (NDP52), that recruit autophagy initiation factors like Unc-51-like kinase 1 (ULK1) and interact with LC3 to form autophagosome (Swerdlow and Wilkins, 2020).

Receptor-mediated mitophagy is mediated by mitophagy receptors located on the outer mitochondrial membrane, like Bcl-2 interacting partner 3 (BNIP3), Nip3-like protein X (NIX), and FUN14 domain-containing protein 1 (FUNDC1), that contain LC3 interacting regions (LIR) motifs (Swerdlow and Wilkins, 2020). Briefly, BNIP3 promotes mitochondrial fragmentation by inhibiting Opa1 and up-regulating Drp1, as well as recruiting Parkin to mitochondria. Parkin in turn ubiquitinates NIX that recruits NBR1 to mitochondria, thus promoting mitophagy (Wang et al., 2023). Both BNIP3 and NIX are up-regulated by hypoxia-inducible factor 1 *α* (HIF-1α), while another mitophagy receptor, FUNDC1, is not subjected to transcriptional regulation by HIF-1α, being regulated by its phosphorylation. Dephosphorylation of FUNDC1 due to hypoxia-induced inactivation of Src kinase results in higher FUNDC1 affinity to LC3, and mitophagy (Ding and Yin, 2012).

Through regulation of mitochondrial elimination, mitophagy is involved in regulation of apoptosis, inflammation, cell differentiation and embryonic development (Onishi et al., 2021). Mitophagy is also essential for neuronal health, although its overactivation may have adverse effects due to excessive reduction of mitochondrial number (Doxaki and Palikaras, 2021).

Impaired mitophagy may be involved in development of neurodegenerative diseases including AD, PD, HD, and amyotrophic lateral sclerosis (Martinez-Vicente, 2017), as well as acute brain damage in stroke, neonatal hypoxic encephalopathy, traumatic brain injury, and epilepsy (Zhang et al., 2021). Therefore, mitophagy may be considered a potential target for development of neuroprotective strategies (Song et al., 2022) or for pathogenesis of various diseases induced by exposure to environmental pollutants including metals (Martínez-García and Mariño, 2020).

### 4.2 Altered brain mitochondrial dynamics and mitophagy upon cu overexposure

Cu-induced alterations in mitochondrial electron transport chain functioning were also associated with impaired mitochondrial dynamics (Table 1). Specifically, a significant reduction in hypothalamic activity of electron transport chain complexes I, II, III, and IV, with a subsequent reduction in mitochondrial oxygen consumption and depletion of ATP production in Cu-exposed pigs, were associated with impaired mitochondrial dynamics and AMP-activated protein kinase (AMPK)-mammalian target of rapamycin (mTOR) pathway activation, altogether contributing to neuronal apoptosis in pig hypothalamus (Lei et al., 2021). Correspondingly, mitochondrial dysfunction, characterized by down-regulated translocase of inner mitochondrial membrane 23 (Timm23) and mitochondrial transcription factor A (TFAM) expression, and a reduction in mitochondrial fusion protein MFN2 expression, together with oxidative stress and neuroinflammation, underlie neuronal damage in cerebral cortex in C57BL/6 J mice induced by CuONPs exposure (Zhou et al., 2021). Furthermore, phosphoproteomic analysis of hippocampus of mice exposed to low-dose Cu revealed significant dysregulation of phosphoproteins associated with mitochondrial functioning and dynamics. Specifically, the authors revealed reduction of phosphoproteins involved in mitochondrial energy metabolism (pyruvate dehydrogenase E1 subunit alpha 1, Pdha1) and mitochondrial biogenesis (protein phosphatase 3 catalytic subunit alpha, Ppp3ca). On the other hand, phosphosites of proteins involved in pathogenesis of AD, BCL2 associated agonist of cell death (Bad) and ubiquinol-cytochrome c reductase core protein 1 (Uqcrc1) were up-regulated. These alterations were associated with down-regulation of mitochondrial biogenesis-related PGC-1α protein expression, suppression of mitochondrial fusion proteins OPA1, MFN1, MFN2 expression, up-regulated expression of mitochondrial fission proteins DRP1 and FIS1, as well as markers of mitochondrial dysfunction, including increased ROS production, reduced ATP levels and cytochrome c oxidase activity (Chen et al., 2019).

**Table 1:** A brief summary of studies demonstrating alterations in brain mitochondrial fusion and fission upon Cu exposure.

Model	Exposure	Dynamics	Other effects	Reference
Weaned pigs	Dietary exposure at 125 and 250 mg/kg Cu for 80 days	↓OPA1 mRNA and protein ↑DRP mRNA	*Hypothalamus* neuronal shrinkage, white matter vacuolization Apoptosis ↑caspase 3, cytochrome c, Bax protein Mitochondrial dysfunction ↓ respiratory chain complex II, III, and IV activity ↓ATP ↓mTOR and AMPK mRNA and protein ↑p-AMPK protein	Lei et al. (2021)
C57BL/6 J male mice	Intratracheal instillation with 30–100 μg/animal CuoNPs	↓MFN2 protein	*Cerebral cortex* Neuronal loss, vacuolar degeneration Neuroinflammation ↑Il-6 and Tnf mRNA Oxidative stress ↑Heme oxygenase (Hmox-1) and thioredoxin (Txn1 and Txn2) mRNA and protein ↓ glutamatecysteine ligase modifer (Gclm) mRNA ↓ p62 protein Mitochondrial dysfunction ↓Tim23 and mitochondrial transcription factor A (TFAM) protein	Zhou et al. (2021)
3xTg-AD and wild-type (WT) mice	Oral exposure to 0.13 ppm Cu with drinking water for 2 months	↑Fis1, DRP1 protein ↓OPA1, Mfn1/2 protein	*Hippocampus* Dysregulated expression of proteins involved in autophagy regulation; synaptic function; neuron function; transcriptional regulation; cytoskeleton; GTPases function; mitochondrial function; energy metabolism; protein kinase; mitochondrial dynamics Mitochondrial dysfunction ↓ mtDNA, ATP level, CytC activity ↓PGC-1a, Nrf1/2, Tfam protein Oxidative stress ↑H2O2, LPO Synaptic damage ↓PSD93, Syn1, synaptophysin protein Axonal degeneration	Chen et al. (2019)
Hy-line chickens	Dietary exposure to 300 mg/kg feed CuSO_4_ and/or 1.25 mg/kg BW As_2_O_3_ for 4–12 weeks	↓Mfn1/2, OPA1 mRNA and protein ↑ DRP1 mRNA and protein ↑↑ upon Cu + As exposure	Moderate neuronal damage, gliosis ↑↑ upon Cu + As exposure chromatin condensation, karyopycnosis mitochondrial swelling and vacuolation autophagosome formation ↑↑ upon Cu + As exposure ↓SOD activity ↑MDA level ↑LC3-II/LC3-I, ATG4B, ATG5, Beclin1, Dynein mRNA ↓TORC1 mRNA	Liu et al. (2020)
Hy-line chickens	Dietary exposure to 300 mg/kg feed CuSO_4_ and/or 2.5 mg/kg BW As_2_O_3_ for 12 weeks	↓Mfn1/2, OPA1 mRNA ↑DRP1 mRNA ↑↑ upon Cu + As exposure	*Cerebrum, cerebellum, brainstem* Mitochondrial swelling, cristae disorganization ↑↑ upon Cu + As exposure Apoptosis chromatin condensation ↑Bax/Bcl-2, Cyt c, p53, Caspase-3 mRNA and protein ↑↑ upon Cu + As exposure ↑IL-2, IL-8, IL-17 mRNA ↓IFN-*γ*, IL-4, IL-10 mRNA ↑↑ upon Cu + As exposure ↑HSP70, HSP90 mRNA and protein	Wang Y. et al. (2020)
Chickens	Dietary exposure to 300 mg/kg feed CuSO4 and/or 2.5 mg/kg BW As_2_O_3_ for 12 weeks	↓Mfn1/2, OPA1 mRNA and protein ↑DRP1 mRNA and protein	*Thalamus* Hemorrhage, inflammatory cell infiltration around local small blood vessels, local tissue necrosis Apoptosis nuclear condensation, chromatin aggregation, and cell volume shrinkage ↑p53, Bax mRNA and protein ↓Bcl-2 mRNA and protein ↑↑ upon Cu + As exposure ↓SOD, Catalase, T-AOC activity ↑MDA level ↑TNFa, iNOS, NF-kB mRNA and protein	Wang D. et al. (2020)
Weanling pigs	Dietary exposure to 125–250 mg/ kg CuSO_4_ for 80 days	↓MFN1/2 protein ↓Opa-1 mRNA (trend) ↑DRP-1 mRNA and protein (trend)	*Cerebrum* Reduction of neurofibrils, vacuolar degeneration Autophagy ↑PINK1, Parkin, and Beclin1, LC3-II mRNA and protein ↓P62 mRNA and protein	Li et al. (2023)
Rats	Intragastric administration of 20–160 mg/kg Cu (as tribasic copper chloride) 12 weeks	Cortex and hippocampus ↑MFN1 protein DRP1 increased and then decreased with exposure time and dose	Memory deficits *Cortex and hippocampus* Neuronal loss, gliosis, neuronal edema, vascular dilatation Mitochondrial vacuolation and swelling, number of mitophagosomes first increased and then decreased with increased Cu exposure NMDA-2A mRNA and protein increased and then decreased with increasing Cu dose aberrant expression of genes involved in regulation of synaptic function, phagosome, and endocytosis LC3B, PINK1, and PARKIN in cerebral cortex and hippocampus increased and then decreased with increasing Cu dose	Yu et al. (2023)

Several studies demonstrated that Cu exposure may aggravate alterations in mitochondrial dynamics induced by other neurotoxic agents. Specifically, Cu significantly potentiates arsenic (As)-induced mitochondrial swelling and vacuolation as well as alterations mitochondrial dynamics by down-regulating fusion-associated MFN1, MFN2, and OPA1 mRNA and protein levels, and increasing the expression of DRP1 in chicken brain (Liu et al., 2020). These alterations were associated with progressive mitochondrial degeneration with mitochondrial swelling, cristae disorganization, and a reduction in mitochondrial number, altogether leading to activation of mitochondrial pathway of apoptosis with increased cytochrome c leakage, increased Bax/Bcl2 ratio, and caspase 3 activation (Wang Y. et al., 2020). Furthermore, Cu and/or As-induced mitochondrial dynamics disorder in chicken thalamus was associated with inflammatory response due to NF-κB activation (Wang D. et al., 2020), in agreement with the role of NF-κB in modulation of mitochondrial dynamics (Carrà et al., 2022).

Cu-induced alterations in mitochondrial functioning, including impaired fusion and fission processes are tightly associated with induction of mitophagy. Specifically, Cu-induced vacuolar degeneration in pig cerebrum was associated with altered mitochondrial dynamics by down-regulating MFN1 and MFN2 mRNA and protein expression, as well as p62 mRNA expression. Impaired mitochondrial dynamics was associated with up-regulation of PINK1, Parkin, and DRP1 mRNA and protein expression indicative of increased mitophagy (Li et al., 2023). Endoplasmic reticulum stress was also observed in association with mitochondrial swelling and mitophagy in cerebrum of chicken exposed to Cu (Huo et al., 2023).

Induction of mitophagy in neuronal cells through a ROS-dependent mechanism upon Cu exposure has been shown to have a protective effect aimed at improvement of mitochondrial dynamic dysregulation, whereas its inhibition by mitophagy inhibitor (Mdivi-1) significantly aggravates Cu-induced neurotoxicity (Zhu et al., 2023). Concomitantly, a detailed study by Yu et al. (2023) demonstrated that exposure to medium (80 mg/kg) and high doses (160 mg/kg) tribasic copper chloride induced neuronal damage in both hippocampus and cortex, that were associated with mitochondrial damage characterized by mitochondrial vacuolation and swelling, as well as altered mitochondrial dynamics and mitophagy. Specifically, exposure for 6 weeks to tribasic copper chloride down-regulated mRNA expression of fusion genes (MFN1/2) and increased fission-related DRP1 mRNA expression, whereas at later stages of exposure (12 weeks), opposite effects were observed, characterized by up-regulated expression of fusion genes and a decrease in fission gene expression both in cerebral cortex and hippocampus. Although mitophagy was up-regulated by both medium and high doses of tribasic copper chloride at 6 weeks of exposure, after 12 weeks of exposure to high doses of Cu mitophagy was inhibited as evidenced by down-regulation of LC3B, PINK1, and PARKIN mRNA expression in both hippocampus and cerebral cortex. In agreement with the proposed role of mitophagy as a stress response to Cu exposure, these findings demonstrate that inhibition of mitophagy by high-dose Cu exposure aggravated mitochondrial dynamics disorder and contributes to cognitive dysfunction (Yu et al., 2023).

It has been also demonstrated that antioxidant 1 copper chaperone (Atox1), trafficking Cu from its transporter Ctr1 to Cu-transporting ATPase, ameliorated neuronal oxidative stress and apoptosis in a mouse model of traumatic brain injury and stretch injury model of HT-22 cells through up-regulation of mitophagy. These effects of Atox1 were mediated by its interaction with DJ-1 and proper Cu binding site, as mutations of Cu-binding motif reduced neuroprotective effects of Atox1 (Zhao J. et al., 2024; Zhao P. et al., 2024).

Cu-induced alterations in mitophagy may contribute to neurodegeneration. Specifically, Cu-induced mitochondrial dysfunction and inhibition of mitophagy, evidenced by down-regulated LC3BII/I, Pink1, and Parkin protein expression, is associated with overexpression of *α*-synuclein (α-syn) in substantia nigra of the exposed mice. The authors proposed that increased α-syn expression may disturb mitochondrial functioning, while mitochondrial dysfunction and impaired mitophagy further aggravates α-syn overaccumulation (Chen et al., 2023).

In addition to the cytotoxic effects of Cu in neurons, Cu-induced microglial activation is also at least partially mediated by mitochondrial membrane damage and impaired mitophagy evidenced by down-regulated Parkin and PINK1 expression in parallel with up-regulation of P62 expression and LC3B-II/I ratio. Combined with ROS overproduction and NF-κB pathway activation, these effects contribute to pyroptosis through up-regulation of NLR family pyrin domain containing 3 (NLRP3)/caspase1/gasdermin D (GSDMD) pathway, whereas NF-κB pathway inhibitor and mitophagy inducer ameliorated proinflammatory effects of Cu (Zhou et al., 2022).

**Figure 1:**
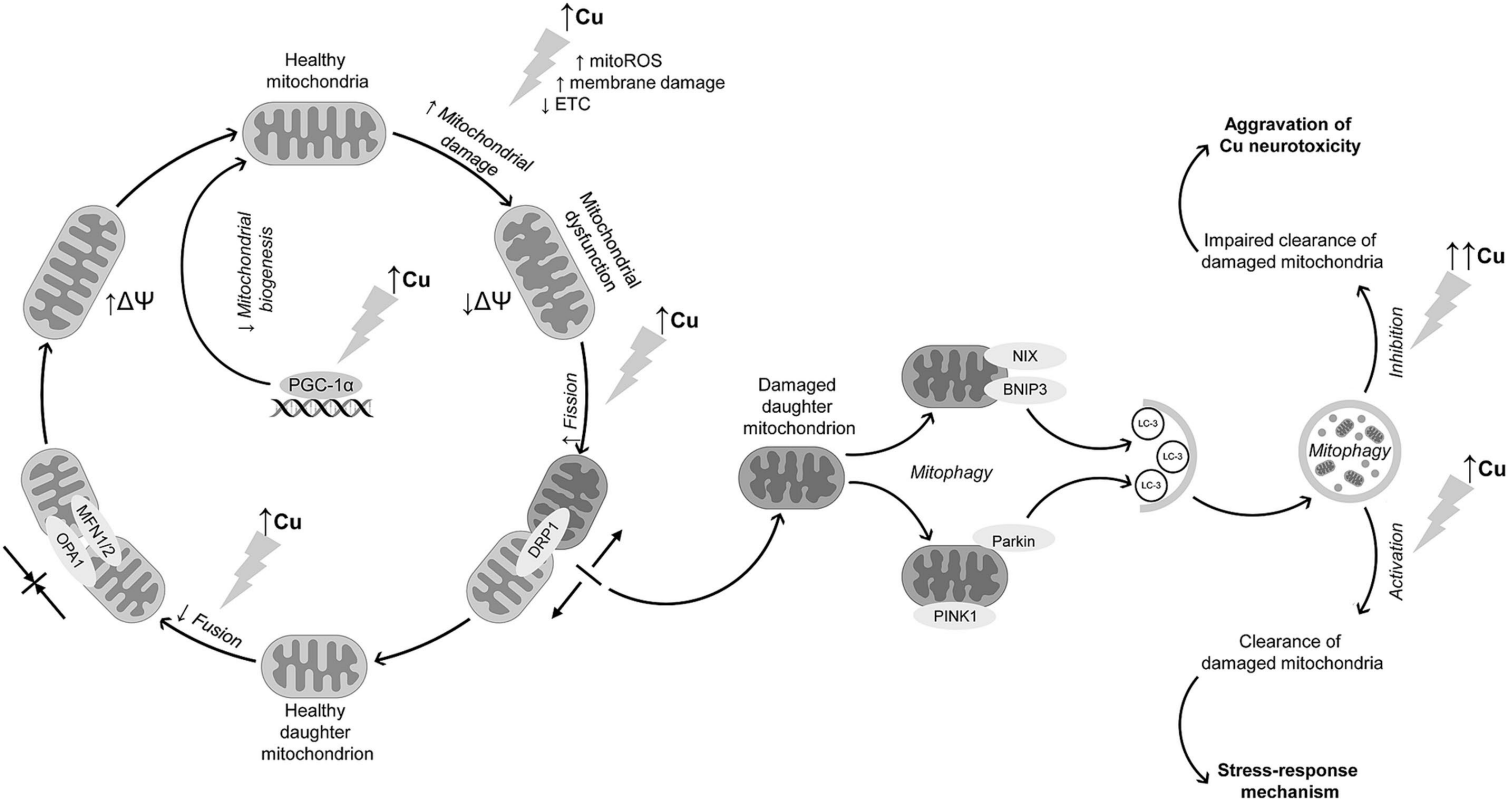
Interference of Cu exposure with mitochondrial dynamics and mitophagy in brain cells. Cu exposure induces mitochondrial dysfunction through ROS overproduction, membrane damage, and ETC inhibition, altogether resulting in a decline in mitochondrial membrane potential. Cu exposure also promotes mitochondrial fission through DRP1 up-regulation. In contrast, mitochondrial fusion is suppressed due to down-regulation of MFN1, MFN2, and OPA1 expression. In parallel with increased mitochondrial fission, Cu exposure promotes neuronal mitophagy through PINK1/Parkin-mediated mechanism, although data from non-neuronal cells demonstrate that receptor-mediated mechanism may be also involved in Cu-induced mitophagy. The latter is considered a stress-response to Cu-induced mitochondrial dysfunction aimed at clearance of damaged mitochondria. Despite activation of mitophagy, long-term high-dose Cu exposure may dysregulate mitophagy, resulting in accumulation of damaged mitochondria and thus aggravation of Cu-induced neurotoxicity. In addition to mitochondrial dysfunction and alterations in mitochondrial quality control mechanisms, Cu overexposure also reduces mitochondrial biogenesis through down-regulation of mitochondrial biogenesis regulator PGC-1α.

Taken together, the existing data demonstrate that in addition to inhibition of electron transport chain and subsequent mitochondrial dysfunction in brain cells, Cu exposure also significantly affects mitochondrial dynamics. This is accomplished by inhibiting mitochondrial fusion through down-regulation of OPA1, MFN1, and MFN2 expression and promoting DRP1-mediated fission (Figure 1). These alterations are also accompanied by Cu-induced activation of mitophagy through up-regulation of PINK1/PARKIN signaling. Mitophagy is considered a protective mechanism in response to mitochondrial stress, being upregulated upon Cu exposure. However, evidence shows that excessive Cu exposure dysregulates mitophagy, thus aggravating Cu-induced mitochondrial dysfunction and neuronal damage. Furthermore, impaired mitophagy may at least partially contribute to microglial activation and neuroinflammation upon Cu exposure.

### 4.3 Evidence from non-neuronal cells on the role of altered mitochondrial dynamics and mitophagy in cu toxicity

Although the present review is focused on discussion of the role of Cu-induced alterations in mitochondrial dynamics and mitophagy in brain cells, studies in non-neuronal cells have also addressed the impact of Cu exposure on mitochondrial quality control mechanisms that may provide additional contribution to understanding of the role of Cu in modulation of mitochondrial pathways. Below, we briefly discuss the contribution from various organ systems.

#### 4.3.1 Liver

Several studies demonstrating the role of Cu exposure in dysregulation of mitochondrial quality control mechanisms were performed in liver *in vivo* or using hepatocytes *in vitro*. Specifically, oral exposure of mice to the increasing doses of Cu not only decreased respiratory chain complexes I-IV content and altered mitochondrial dynamics with down-regulated MFN1/2 and up-regulated DRP1 and FIS1 expression, but also impaired hepatic expression of PGC-1α, TFAM, and nuclear respiratory factor 1 (NRF-1) involved in mitochondrial biogenesis (Wang et al., 2024; Wang et al., 2024a,b). Induction of mitochondrial dysfunction and impaired mitochondrial dynamics with down-regulated MFN1/2 and OPA1 expression in parallel with enhanced fission-related DRP1 expression were shown to contribute to liver damage and growth inhibition induced by long term Cu exposure in weanling pigs (Hu et al., 2023). Cu-induced mitochondrial fission in primary chicken embryo hepatocytes was also associated with up-regulation of mitochondrial fission regulator 1 (Mtfr1), mitochondrial fission process protein 1 (Mtfp1), and dynamin 1 (Dnm1) mRNA expression in a dose-dependent manner (Wu et al., 2021). Taken together, similarly to brain cells, studies using liver cells demonstrated that altered mitochondrial dynamics is associated with up-regulation of DRP1 with a concomitant down-regulation of MFN1/2 and OPA1 expression. However, findings from hepatocytes also highlight that Cu may affect mitochondrial dynamics through modulation of Mtfr1, Mtfp1, and Dnm1 mRNA expression, thus indicating the potential involvement of these mechanisms in Cu-induced alterations of mitochondrial dynamics in brain.

The protective effect of mitophagy against Cu toxicity was also demonstrated in chicken liver, where induction of mitophagy by rapamycin significantly attenuated Cu-induced mitochondrial apoptosis, while autophagy inhibitor 3-methyladenine aggravated Cu-induced apoptosis and S-phase arrest in cell cycle (Yang D. et al., 2021; Yang F. et al., 2021). Correspondingly, activation of mitophagy was observed in Cu-exposed ATP7B-deficient hepatocytes characterized by Cu overload, a model of WD, whereas PINK1 and BNIP3L depletion promoted cellular apoptosis, thus supporting the role of mitophagy as a protective mechanism in ATP7B-deficient cells (Polishchuk et al., 2019). Finally, a novel mitochondria-targeting Cu complex containing tri-phenyl-phosphonium groups ([Cu(ttpy-tpp)Br2]Br) inhibited aerobic glycolysis, induced mitochondrial dysfunction, promoted DRP1-mediated mitochondrial fission, and induced mitophagy through the release of hexokinase-2 from mitochondria in human hepatoma cells (Li et al., 2020). Although Cu supplementation was capable of inducing mitophagy in rat liver by up-regulating PINK1, PARKIN, beclin 1 (BECN1) and LC3B-II/I protein expression with a concomitant increase in mitophagosome number, hepatic Cu accumulation upon exposure to high doses of Cu impaired mitophagy and promoted hepatocyte apoptosis (Yu et al., 2021). Therefore, findings from hepatocytes also support the role of mitophagy as a stress-response mechanism to Cu overload, as observed in brain cells, while impairment of this mechanism upon exposure to high doses of Cu may aggravate toxic effects of Cu. Such an effect may be observed in WD, when both liver and brain accumulate excessive levels of Cu.

The most recent studies demonstrated that non-coding micro RNAs may be involved in regulation of Cu-induced alterations in mitochondrial dynamics. Specifically, Cu-induced mitochondrial ROS production, reduction of mitochondrial fusion and excessive fission, inhibition of mitochondrial biogenesis, and aberrant mitophagy in chicken liver, were mediated by up-regulation of mitomiR-12294–5p expression leading to CDGSH iron–sulfur domain-containing protein 1 (CISD1) inhibition, as mitomiR-12294–5p inhibition significantly reduced adverse effects of Cu on mitochondrial oxidative stress and dynamics (Zhong et al., 2023). Although the role of epigenetic mechanisms in Cu-induced alterations of mitochondrial dynamics were not observed in brain cells, these findings demonstrate that modulation of non-coding RNA that is observed in brain upon Cu exposure (Hsu et al., 2019) may also contribute to impaired mitochondrial quality control and Cu neurotoxicity. However, the role of mitochondrial miRNA in Cu-induced mitochondrial dysfunction and brain damage and is yet to be estimated.

#### 4.3.2 Cardiovascular system

Alterations of mitochondrial dynamics and mitophagy following Cu exposure were also revealed in cardiovascular system. Specifically, protein–protein interaction analysis using STRING 10 functional protein association networks website demonstrated that upon Cu exposure mitochondrial fission-associated gene Drp1 is coexpressed with caspase 8 and cytochrome c genes, whereas expression of Mfn1/2 is tightly interrelated with Bcl-2 expression in chicken hearts (Li et al., 2017). Correspondingly, mitochondrial fission in EA.hy926 vascular endothelial cells exposed to CuONPs also induced PINK1-mediated mitophagy, whereas Tax1 binding protein 1 (TAX1BP1) plays an essential role in linking of ubiquitinated mitochondria to autophagosomes (Fan et al., 2022). In turn, CuONPs-induced mitochondrial dysfunction with overproduction of superoxide triggers mitophagy in vascular endothelial cells as evidenced by colocalization of LC3B and translocase of outer mitochondrial membrane 20 (TOM20), while superoxide detoxication ameliorated formation of mitophagosomes. At the same time, lack of degradation of ATP5B and TOM20 suggests that mitophagic flux appears to be impaired upon CuONPs exposure due to lysosomal dysfunction, thus leading to accumulation of damaged mitochondria and increased superoxide levels, contributing to cell death (Zhang et al., 2018). Although these findings corroborate the earlier discussed effects of Cu on mitophagy in brain cells, data from cardiovascular system highlight the potential role of TAX1BP1 and TOM20 in Cu-induced modulation of mitophagy that were not detected yet in brain upon Cu exposure.

#### 4.3.3 Gastrointestinal tract

In addition to liver, it has been demonstrated that Cu exposure may impair mitophagy in other organs of gastrointestinal system including intestinal epithelium (Ma et al., 2022). It is also assumed that Cu-induced alterations in gut microbiota may contribute to endoplasmic reticulum stress, mitophagy and apoptosis in duodenum of chicks. Although the most prominent correlations were observed between gut microbial taxa and endoplasmic reticulum stress-related genes, expression of PINK1 directly correlated with the relative abundance of *Lactococcus*, while the highest correlation coefficients for the association with autophagy protein 5 (Atg5) expression were observed for the abundance of *Melainabacteria*, *Ruminococcaceae*, *Eggerthellaceae* and *Bacteroidaceae*. In turn, LC3-I and LC3-II were negatively associated with the numbers of *Lactococcus* (Ma et al., 2022). It has been also demonstrated that up-regulation of miR-1285 in jejunal epithelium may contribute to adverse effects of Cu on mitochondrial functioning and mitophagy through inhibition of isocitrate dehydrogenase 2 (IDH2) expression (Liao et al., 2022). It has been also demonstrated that alterations in mitochondrial dynamics in pig fundic gland upon Cu exposure, characterized by down-regulated MFN1, MFN2, and OPA1 mRNA and protein expression and increased DRP1 expression, are associated with mitochondrial unfolded protein response evidenced by up-regulated heat shock protein 60 (HSP60), HSP10, C/EBP homologous protein (CHOP), HtrA serine peptidase 2 (HTRA-2), and CLPP mRNA expression (Huo et al., 2021). These findings demonstrate that endoplasmic reticulum stress induced by Cu exposure may promote dysregulation of mitochondrial dynamics and mitophagy. Given the earlier demonstrated role of ERS in Cu-induced neurotoxicity (Tanaka and Kawahara, 2017; Huo et al., 2023), it is assumed that this mechanism may be also interrelated with Cu-induced mitochondrial dynamics dysregulation in brain. In addition, studies using intestinal epithelium also support the potential role of miR as regulators of mitophagy upon Cu exposure.

#### 4.3.4 Other tissues

The role of altered mitochondrial dynamics and mitophagy as a potential mechanism was also demonstrated in cells of other systems. Specifically, an *in vitro* study demonstrated that in duck renal tubular epithelial cells Cu exposure induced severe mitochondrial damage, reduced mitochondrial membrane potential, ATP content, mitochondrial fusion but enhanced fission. There was also down-regulation of mitochondrial biogenesis-associated PGC-1α expression, suggesting mitophagy as a stress response. In addition to up-regulated expression of LC3A, LC3B, and P62, Cu-induced mitophagy was associated with overexpression of other mitophagy-associated genes, including BNIP3, NIX, OPTN, NDP52, lysosomal-associated membrane proteins 1 (LAMP1) and LAMP2. On the other hand, inhibition of mitophagy with cyclosporine A aggravated Cu-induced mitochondrial dysfunction (Bai et al., 2023). It has been demonstrated that, in HEK293 cells, induction of mitophagy in response to Cu-induced oxidative stress is at least partially dependent on Sestrin2 association with mitochondrial protein ATP5A through C-terminal domain and that its phosphorylation by ULK1, being indicative of the role of ULK1-Sestrin2 pathway as a mechanism of stress response to Cu toxicity (Kim et al., 2020). Therefore, these findings demonstrate that mitophagy is a critical mechanism of protection from Cu toxicity, and its induction may be assisted not only by p62, as demonstrated in brain cells, but also OPTN and NDP52, as well as BNIP3/NIX during ubiquitin-independent mitophagy. Furthermore, studies using kidney cells highlighted the role of ULK1-Sestrin2 pathway in mitophagy induction. Hypothetically, these mechanisms may be also involved in Cu-induced dysregulation of mitophagy in brain cells, although their particular contribution to Cu neurotoxicity requires further confirmation.

Single studies highlighted the role of altered mitochondrial dynamics in Cu-induced damage in testis, spleen, and lungs. Specifically, in testis of Cu-exposed chicken, a significant association between Mfn1/2 and Bcl-2, Bax, ATG5, LC3-I and LC3-II expression was revealed, suggesting of a tight relationship between Cu-induced alterations in mitochondrial fusion, apoptosis, and autophagy (Shao et al., 2019). A study by Wang et al. (2018) demonstrated that in chicken skeletal muscle, Cu-induced alterations in mitochondrial dynamics and induction of mitophagy may be mediated by inhibition of phosphoinositide 3-kinase (PI3K)/AKT/mTOR pathway (Wang et al., 2018). In spleen of Cu-exposed pigs, correlation analysis demonstrated a strong inverse relationship between expression of mitochondrial fusion proteins, MFN2 and to a lesser extent MFN1 and OPA1, and proteins involved in endoplasmic reticulum stress and apoptosis, indicative of a tight association between these mechanisms (Zhang K. et al., 2023; Zhang Y. et al., 2023). Despite the previously discussed role of Cu exposure in alteration of mitochondrial dynamics, it has been demonstrated that non-cytotoxic doses of Cu significantly improved mitochondrial bioenergetics and turnover, as well as increased proliferation and differentiation of K562 cells (Ruiz et al., 2016). In addition, intratracheal instillation with CuONPs in mice resulted in lung damage, and was associated with activation of mitophagy with a dose-dependent up-regulation of LC3B, p62, ULK1, Beclin1, and ATG protein expression in lung tissue. In turn, mitophagy disorder induced by LC3B (lc3b^−/−^) deficiency aggravated lung injury due to accumulation of damaged mitochondria overloaded with Cu ions, further supporting the hypothesis of protective effect of mitophagy against Cu toxicity (Xiao et al., 2021).

The association between Cu exposure and altered mitochondrial dynamics was also confirmed in fish studies. Specifically, it was demonstrated that up-regulation of pre-hypoxia-induced up-regulation of Forkhead box O3 (FoxO3) gene expression in Cu-exposed large yellow croaker *Larimichthys crocea* is significantly associated with an increase in LC3a, PINK1, Parkin, Nix, and Mfn2 mRNA expression, indicative of the role of Cu in modulation of mitochondrial fusion and mitophagy (Zeng et al., 2020). A study in rabbitfish *Siganus fuscescens* demonstrated that mitophagy is involved in removal of excessive Cu accumulated in mitochondria, promoting Cu transfer from mitochondrial fraction to lysosomes and its subsequent excretion (Xia and Wang, 2023).

Taken together, data from non-neuronal cells corroborate findings from brain cells demonstrating Cu-induced alterations in mitochondrial dynamics and mitophagy, but also highlight additional intimate mechanisms that may contribute to impaired mitochondrial quality control in brain upon Cu exposure. Specifically, these findings highlighted that Cu may induce mitophagy not only via non-receptor-mediated (PINK1/Parkin-dependent) mitophagy as observed in brain cells, but also through activation of receptor mediated mitophagy involving NIX, BNIP3, ULK1, and Sestrin2 signaling. In addition, it is evident from non-neuronal cells that Cu-induced dysregulation of mitochondrial dynamics and mitophagy may be modulated by induction of endoplasmic reticulum stress, epigenetic mechanisms including modulation of miRNA expression, as well as alterations in gut microbiota. However, the contribution of these mechanisms to Cu-induced disturbances in mitochondrial pathways in neural cells has yet to be clarified.

## 5 Cuproptosis as a novel cu-induced cell death mediated by mitochondrial proteotoxic stress

**Figure 2:**
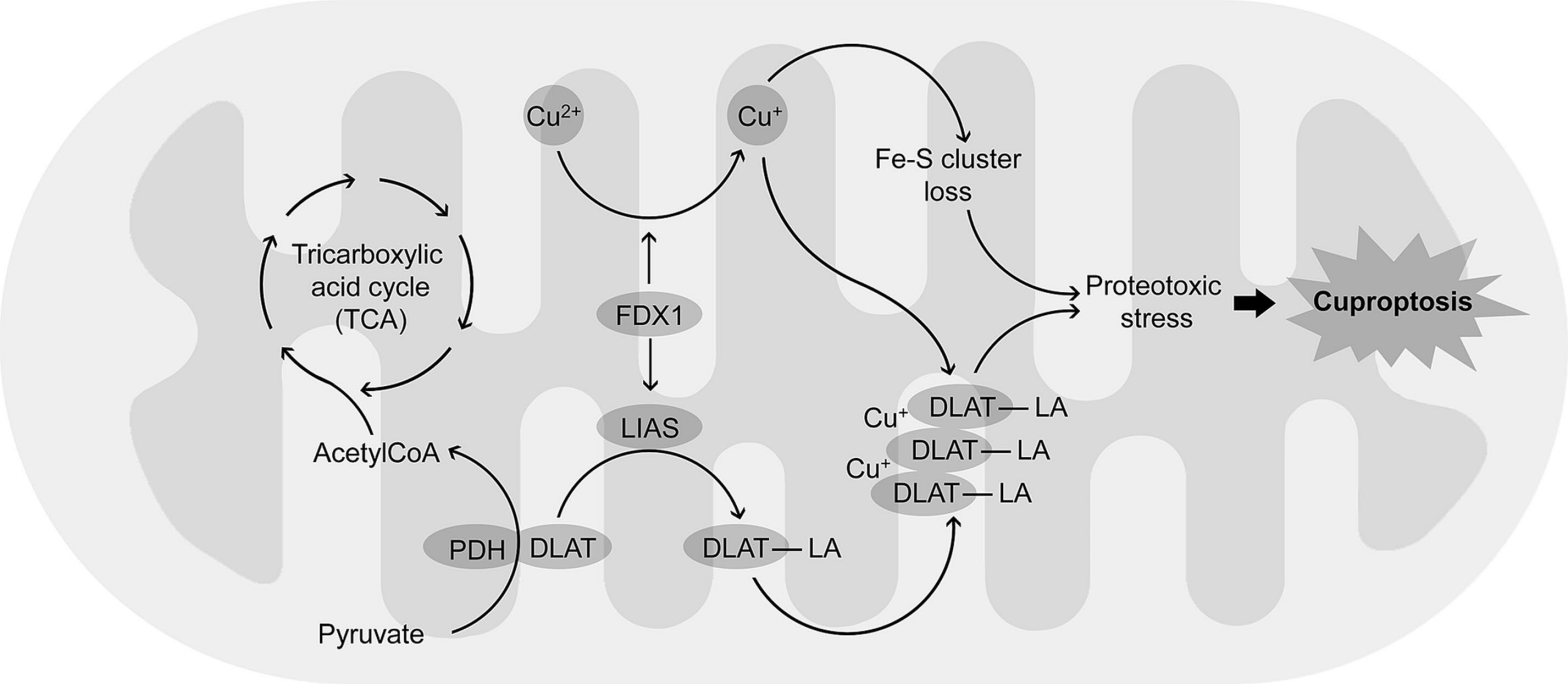
Mechanisms of cuproptosis, a Cu-dependent form of programmed cell death. Cu2+ accumulating in mitochondria is reduced to a more toxic Cu + by FDX1. FDX1 also promotes lipoylation of proteins including DLAT by up-regulation of LIAS. Binding Cu + to lipoylated DLAT (DLAT-LA) induces protein aggregation. The latter results in proteotoxic stress that is also associated with Cu-induced loss of Fe-S cluster proteins, altogether leading to cuproptosis.

While discussing the role of mitochondrial dysfunction in Cu neurotoxicity, it is essential to address a recently discovered form of cell death, cuproptosis (Tsvetkov et al., 2022), that is dependent on intracellular Cu trafficking and mitochondrial functioning (Tian et al., 2023). This type of Cu-triggered mitochondrial cell death is different from oxidative stress-driven types of cell death like apoptosis, necroptosis, or iron-induced ferroptosis (Tang et al., 2022). Briefly, after entering mitochondria, Cu^2+^ is reduced to a more toxic Cu^+^ by ferredoxin (FDX1). The latter also acts as a regulator of protein lipoylation (Tian et al., 2023) by direct binding to lipoyl synthase (LIAS) (Dreishpoon et al., 2023). Binding of Cu^+^ to lipoylated tricarboxylic acid cycle proteins, and specifically dihydrolipoamide S-acetyltransferase (DLAT), leads to its oligomerization (Wang et al., 2024; Wang et al., 2024a,b). In addition, Cu induces a loss of Fe-S cluster proteins hypothetically through replacing iron or its oxidation, as well as oxidative damage (Lou et al., 2024). Together with proteotoxic stress induced by lipoylated proteins aggregation, Cu-induced reduction of Fe-S stability contributes to cell death termed cuproptosis (Figure 2).

An increasing body of data demonstrate that cuproptosis plays a significant role in neuronal damage and development of brain diseases, including neurodegeneration (Song et al., 2024). Although it is assumed that cuproptosis is one of the potential mechanisms of Cu neurotoxicity, the direct evidence supporting this hypothesis is scarce (Feng et al., 2023).

It has been demonstrated that Cu overexposure induced cognitive dysfunction in mice by increasing hippocampal neuron death not only through apoptosis, but also cuproptosis, as evidenced by up-regulation of DLAT protein expression along with suppression of Fe-S cluster-associated protein (FDX1, DNA polymerase delta 1 catalytic subunit (POLD1), and aconitase 2 (ACO2)) expression. Cu-induced neurotoxicity was also associated with reduced synaptic plasticity with down-regulation of synaptophysin and postsynaptic density protein 95 (PSD-95) expression through inhibition of cAMP response element-binding protein (CREB)/brain-derived neurotrophic factor (BDNF) pathway (Zhang K. et al., 2023; Zhang Y. et al., 2023). In a model of transient middle cerebral artery occlusion (tMCAO), Cu accumulation in brain was associated with mitochondrial swelling and cristae damage. It is proposed that tMCAO results in up-regulated expression of FDX1 that converts Cu(II) to a more reactive Cu(I), resulting in mitochondrial dysfunction, inflammatory response, and cuproptosis. On the other hand, FDX1 inhibition by disulfiram ameliorated neurotoxic effects of Cu (Yang et al., 2024). In turn, reduction of brain Cu levels using dexmedetomidine and/or Cu chelator D-penicillamine resulted in cuproptosis inhibition through improvement of mitochondrial functioning and a significant reduction in LIAS, succinate dehydrogenase complex iron sulfur subunit B (SDHB), DLAT, dihydrolipoamide S-succinyltransferase (DLST), and FDX1 protein expression, all associated with reduction of brain infarction volume in a rat model of in cerebral ischemia/reperfusion (Guo et al., 2023).

Furthermore, bioinformatic analysis also revealed a significant association between cuproptosis-related genes and pathogenesis of a number of brain diseases, including temporal lobe epilepsy (Yang et al., 2023), PD (Zhang H. et al., 2024; Zhang J. Y. et al., 2024; Zhang L. et al., 2024; Wu et al., 2023), ischemic stroke (Qin et al., 2024), and ASD (Zhou and Gao, 2022), to name a few. These data allow to propose a potential role of cuproptosis in neurotoxic effects of Cu overload, although it is still questionable whether cuproptosis pathway will contribute to development of these diseases upon environmental Cu exposure.

Despite the fact that the experimental evidence linking Cu overexposure to cuproptosis in neural cells is scarce, several studies in other cells and tissues support the putative role of cuproptosis as a mechanism of Cu-induced cytotoxicity. Specifically, it has been proposed that Cu overload plays a role in induction of miscarriage in mice due to placental cuproptosis (Zhao et al., 2024). Exposure to the increasing doses of Cu down-regulated mRNA and expression of proteins associated with TCA, while increasing both gene and protein expressions of Lipt1 and Lias. Expression of Fdx1 was also found to be up-regulated in response to Cu exposure, whereas mRNA and protein expression of Fe-S-cluster-associated proteins, Pold1, Cisd1, phosphoribosyl pyrophosphate amidotransferase (Ppat), Sdhb, regulator of telomere elongation helicase 1 (Rtel1), Nth like DNA glycosylase 1 (Nthl1), and DNA polymerase epsilon catalytic subunit (Pole), were decreased. Furthermore, the authors demonstrated that Cu-induced cuproptosis in mouse placenta may be epigenetically regulated. It was suggested that Cu exposure down-regulates mmu-miR-3473b expression that contributes to inhibition of Dlst and Rtel1 mRNA expression, while mmu-miR-3473b overexpression significantly reduces cytotoxic effects of Cu^2+^ (Zhao et al., 2024). Correspondingly, in trophoblast cells Cu exposure, especially with elesclomol, a mitochondrion-targeting copper ionophore developed as a chemotherapeutic agent (Tarin et al., 2023), induced cuproptosis with up-regulation of FDX1, HSP70, and Cu-importing SLC31A1, whereas the proteins involved in mitochondrial functioning (NADH: ubiquinone oxidoreductase subunit B4 (NDUFB4), IDH2, DLAT, and cytochrome C oxidase assembly homolog COX15), Fe-S cluster-associated proteins (RTEL, CISD1, SDHB, PPAT, NTHL1, and POLD1), and Cu-exporting protein ATP7B were down-regulated, that may be at least partially mediated by lnc-HZ11 expression (Zheng et al., 2024).

It has been also demonstrated that cuproptosis is involved in cytotoxic effect of Cu in testicular spermatogenic cells, as an increase in cellular Cu levels after exposure was associated with mitochondrial dysfunction and up-regulation of FDX1, SLC31A1, and DLAT protein expression (Zhang H. et al., 2024; Zhang J. Y. et al., 2024; Zhang L. et al., 2024). Noteworthy, aggravation of ageing-associated testicular damage by Cu exposure in Drosophila may be mediated not only by induction of cuproptosis, but also ferroptosis, while adequate expression of long non-coding RNA (lncRNA) CR43306 may reduce adverse effects of Cu (Huang D. et al., 2024; Huang Q. et al., 2024). Furthermore, it is suggested that cuproptosis upon CuONPs exposure may promote significant dysregulation of redox homeostasis, mitochondrial dysfunction, and altered mitochondrial membrane synthesis, subsequently promoting ferroptosis (Jiang et al., 2024). In turn, Cu chelation significantly reduced cuproptosis both in lipopolysaccharide (LPS)-induced macrophages and in a mouse model of periodontitis through down-regulation of *Fdx1*, *Lias*, *Dld*, and *Lipt1* mRNA expression, as well as promoted mitophagy-related gene expression (Zhang H. et al., 2024; Zhang J. Y. et al., 2024; Zhang L. et al., 2024).

Despite paucity of information, laboratory evidence demonstrate that Cu exposure induces cuproptosis-mediated cell death in brain cells, and bioinformatic processing of human studies supports the role of cuproptosis in brain disease pathogenesis. Studies from non-neuronal also indicate that Cu exposure induce cuproptosis that may be at least partially mediated by epigenetic mechanisms.

## 6 Conclusion

Existing data corroborate that brain mitochondria are a primary target for Cu neurotoxicity. Reduction of mitochondrial membrane potential in neurons and glia, non-neuronal brain cells, following Cu exposure has been shown to be associated with inhibition of electron transport chain complexes, binding to free thiol groups of mitochondrial proteins, mitochondrial ROS production, and membrane damage. Moreover, Cu exposure aggravates mitochondrial toxicity of exogenous toxicants (H_2_S, Pb, As) and amyloid beta.

An increasing body of evidence demonstrates that Cu exposure also interfered with mitochondrial dynamics. Specifically, Cu exposure inhibits mitochondrial fusion through down-regulation of Opa1, Mfn1, and Mfn2, while promoting mitochondrial fission due to up-regulation of Drp1 expression. Such alterations are associated with aggravation of mitochondrial dysfunction, inhibition of electron transport chain, and induction of apoptosis.

Cu-induced induction of mitochondrial dysfunction and promotion of mitochondrial fission is also associated with activation of mitophagy in brain cells through up-regulation of Pink1 and Parkin expression. The latter is considered a compensatory response to Cu-induced mitochondrial damage that removes damaged mitochondria. However, prolonged high dose exposure to Cu may induce mitophagy disorder, thus promoting accumulation of damaged mitochondria. Inhibition of mitochondrial biogenesis following Cu exposure also aggravates brain mitochondrial dysfunction. Indeed, it has been demonstrated that dysregulated mitochondrial dynamics and mitophagy may contribute not only to Cu-induced neuronal damage, but also to microglial activation, thus contributing to neuroinflammation. Studies from non-neuronal cells demonstrate that induction of mitophagy upon exposure to Cu may involve not only non-receptor-mediated (PINK1/Parkin-dependent), but also receptor-mediated (PINK1/Parkin-independent) pathways. These studies have also revealed the potential role of epigenetic mechanisms and gut microbiota in modulation of Cu-induced alterations of mitochondrial dynamics and mitophagy, although the relevance of these mechanisms for brain cells is yet to be elucidated.

Finally, several studies demonstrated that Cu exposure induces brain cell cuproptosis, a type of cell death due mitochondrial proteotoxic stress. Despite the lack of dose–response studies in human populations, bioinformatic analysis also confirms the association between cuproptosis-related genes and neurological disorders. Certain studies from non-neuronal cells corroborate the observed role of Cu overexposure in induction of cuproptosis, as well as demonstrate the potential role of epigenetic mechanisms in this relationship, however, the role of epigenetics in neuronal cuproptosis is yet to be elucidated.

Therefore, strong support exists for mitochondria as a primary target of Cu neurotoxicity. Alterations of mitochondrial dynamics and dysregulation of mitophagy may play a significant role in Cu-induced mitochondrial dysfunction, thus contributing to neuronal death and neuroinflammation. Moreover, activation of cuproptosis following Cu exposure also contributes to neuronal damage. Based on these findings, it is assumed that development of mitoprotective agents, specifically targeting mechanisms of mitochondrial quality control, may be used for prevention of neurotoxic effects of not only Cu overload, but also a plethora of mitophagy-related disorders.
